# Supplementary material for: Predictive Factors and Clinical Impacts of Delayed Isolation of Tuberculosis during Hospital Admission
Source: J Clin Med. 2023 Feb 8;12(4):1361. doi: 10.3390/jcm12041361 (PMC9966369; doi:10.3390/jcm12041361)
Supplement: Supplementary file 1 [file jcm-12-01361-s001.zip › jcm-2175961-supplementary.pdf]

Supplementary File

Table S1. Summary of contact investigation by index patients.

Index No.	Category	Department of admission	Diagnosis	Route of admission	AFB smear/ *Mtb* culture/ Xpert MTB/RIF	Exposure days	Exposure site	No. of HCW close contacts	Initial radiologic findings	Tuberculosis related symptom on day 1	
										Respiratory	Nonrespiratory
1	A	Neurology	Status epilepticus	ER	-/+/+	10	ER	7	con	S, D	F, MC
2	A	Neurosurgery	Intracerebral hemorrhage	ER	-/+/+	1	ER	8	nod	S	F, MC
3	A	Orthopedic surgery	Dislocation of hip	ER	-/-/+	1	ER	6	nod, cal, fib	S	
4	A	Neurology	Status epilepticus	ER	-/+/+*	31	ER, ICU	23	nod, cav, cal, fib, GGO	S	F, MC
5	A	Pulmonology	Pulmonary tuberculosis	ER	-/-/+	0	ER	8	con, fib	S	F, MC
6	A	Gastroenterology	Alcoholic Ketoacidosis	ER	-/-/+	2	ER, ICU	11	normal		A, MC
7	A	General surgery	Panperitonitis	ER	-/+/+	1	ICU, OR	12	con	S	MC
8	B	Pulmonology	Pulmonary tuberculosis	ER	3+/+/-	2	Ward	0	con, GGO	C, S, D	CH, GW, A
9	B	Pulmonology	Pneumonia	ER	-/-/+*	3	Ward, ICU	18	con, GGO	D	F
10	B	Pulmonology	Pulmonary tuberculosis	ER	-/-/+*	12	Ward	3	con, nod, cav, fib, GGO	C, S, D	CH, GW, A
11	B	Pulmonology	Pulmonary tuberculosis	ER	3+/+/-	1	Social work department	1	nod, cav, GGO	C, D	

12	C	Pulmonology	Interstitial lung disease, Chronic obstructive lung disease	OPD	-/-/+*	3	Ward	0	con	D	F
13	C	Pulmonology	Pneumonia	OPD	2+/+/+*	11	Ward	0	con, nod	C, S	
14	D	Nephrology	Acute kidney injury	ER	1+/+/+	3	Ward	0	con, nod		CH, GW
15	D	Nephrology	Chronic kidney disease	OPD	-/+/+	2	Ward	0	con, nod		
16	D	Ophthalmology	Cataract	OPD	1+/+/+	4	Ward, OR	5	con, nod, fib		
17	D	Urology	Prostate cancer	OPD	-/+/+	4	PFT unit	1	con		
18	E	Orthopedic surgery	Femur neck fracture	ER	-/+/+	15	Ward	0	con	S	
19	E	General surgery	Intestinal tuberculosis	ER	-/+/+	13	Ward	0	mass		F, A
20	E	Neurology	Myasthenia gravis	OPD	-/-/+*	20	ICU	5	nod, cal, mass		
21	E	Hematology & oncology	Diffuse large B-cell lymphoma	OPD	2+/+/+	21	Ward	10	con		GW, A
22	E	Neurosurgery	Intracerebral hemorrhage	ER	-/-/+*	5	ICU	12	con	S	F
23	E	Thoracic surgery	Chest wall tuberculosis	OPD	-/-/+	13	OR	8	nod, mass		CP
24	E	Gastroenterology	Gastric cancer	ER	-/+/+	7	ICU, PFT unit, endoscopy unit	16	nod		

25	E	Gastroenterology	Liver cell carcinoma	OPD	1+/+/+	5	Ward, PFT unit	3	nod, cal, fib	C, D

* bronchial washing

ER, emergency room; OPD, outpatient clinic; ICU, intensive care unit; OR, operation room; PFT, pulmonary function test; Con, consolidation; nod, nodule; cav, cavity; cal, calcification; fib, fibrosis; GGO, ground-glass opacity; C, cough; S, abnormal sputum; D, dyspnea; F, fever; CH, chill; MC, mental change; GW, general weakness; A, anorexia; CP, chest pain.

Table S2. Summary of contact investigation by categories.

Category	Reasons of delayed isolation	Number of index patients, n (%)	Exposure days, median (IQR)	Number of exposure events, n (%)	Number of exposure events per index patient, median (IQR)
A	Missed during emergency situation	7 (28.0%)	1.0 (1.0–6.0)	75 (47.8%)	8.0 (7.5–11.5)
B	Positive conversion after serial results	4 (16.0%)	2.5 (1.8–5.3)	22 (14.0%)	2.0 (0.8–6.8)
C	Coexistent respiratory disease	2 (8.0%)	7.0 (5.0–9.0)	0 (0.0%)	0.0 (0.0–0.0)
D	Delayed check of test results	4 (16.0%)	3.5 (2.8–4.0)	6 (3.8%)	0.5 (0.0–2.0)
E	Missed test despite an abnormal chest radiograph	8 (32.0%)	13.0 (6.5–16.3)	54 (34.4%)	6.5 (2.3–10.5)
